# Experimental Study on the Flexural Performance of Parallel Strand Bamboo Beams

**DOI:** 10.1155/2014/181627

**Published:** 2014-02-18

**Authors:** Aiping Zhou, Yuling Bian

**Affiliations:** ^1^School of Civil Engineering, Nanjing Forestry University, Nanjing, Jiangsu 210037, China; ^2^Wuxi Institute of Commerce, Wuxi, Jiangsu 214153, China

## Abstract

Searching for materials to provide proper housing with less emission and low energy becomes an urgent demand with the ever-growing population. Bamboo has gained a reputation as an ecofriendly, highly renewable source of material. Parallel Strand Bamboo (PSB) is a new biocomposite made of bamboo strips which has superiority performances than wood products. It has attracted considerable interests as a sustainable alternative for more traditional building materials. But the mechanical performance study of PSB as construction materials is still inadequate. Also, the structural behavior of PSB is not quite understood as conventional construction materials, which results in the difficulties to predict the performances of PSB structural members. To achieve this purpose, 4-point bending experiments for PSB beams were carried out. The flexural performances, mode of failure in bending, and the damage mechanism of PSB beams were investigated in this paper.

## 1. Introduction

In recent years, with the ever-growing population, searching for materials to provide proper housing with less emission and low energy becomes a challenge. Bamboo has gained a reputation as an ecofriendly, highly renewable source of material. Bamboo grows much faster than wood. Usually could reached maturity in 5 years. But raw bamboo can not meet the requirements as modern building materials because of their many varieties in mechanical properties and in geometrical shapes and sizes. To meet the requirements of current structural applications, many efforts have been made to bamboo to have stable properties and more uniform. Parallel Strand Bamboo (PSB) belongs to one of the engineered materials made with bamboo, which is fabricated by cutting bamboo into strips along parallel-to-grain direction by using adhesive in the lamination process parallel to each other into a prism often with a rectangular cross section [1]. It has attracted considerable interests as a sustainable alternative for more traditional building materials due to its outstanding mechanical performances. Currently, the compressive strength and Young's modulus of PSB had reached more than 60 MPa and 12000 MPa, respectively [2], which could meet the standard as structure materials for buildings. But the mechanical performance study of PSB as construction materials is still inadequate. Also, the structural behavior of PSB is not quite understood as conventional construction materials, which results in difficulty to predict the mechanical performances of PSB. The parameters used in the design of some demohouses in China were obtained by testing of the structural members [3–5]. As a result the use of PSB as construction material is not as common. Originally PSB were only used as floor boards or decoration materials. Its physical and mechanical properties have been studies largely as decoration materials [6]. Hence, the criteria in approaching to use large scale in PSB as construction materials are to establish the design philosophy for PSB structure. This paper reported part of these works which aimed to study the flexural performance of PSB beams. The damage mechanism of PSB beams was investigated by using 4-point bending test.

## 2. Brief of Experiments for Beams

### 2.1. Preparation of PSB Beams

Bamboo for the experiment came from Anhui province in China, which had been grown for 5 years. Bamboo was cut into many segments with a length of 2 m parallel to its grain. To study the mechanical performance of its different parts, three groups of samples were divided into the bottom, the middle, and the top part of the culm. Then every segment was split into strips (3 mm thickness, 10 mm width, and 2 m length). These strips gotten rid of the bamboo green and the bamboo yellow were grinded to be linked together in parallel-to-grain directions and unlinked in transverse-to-grain directions. In order to reduce the starch content, bamboo strips were carbonized in the steam oven with a pressure of 0.3 MPa for 90 min., as bamboo with low humidity is less prone to mould attacks. For its conservation, bamboo was air-dried in a drying house, and then these bamboo strips were soaked in a glue water pond for 10 min., air-dried again in the drying house to a humidity moisture content of about 12%, and loaded into an iron mould for thermoforming in the stove with a temperature of 120°C, at 150 MPa pressure for 8 hours. PSB beams (160 mm × 110 mm × 1880 mm) were fabricated by this method.

### 2.2. Experimental Design of PSB Beams

**Figure 1:**
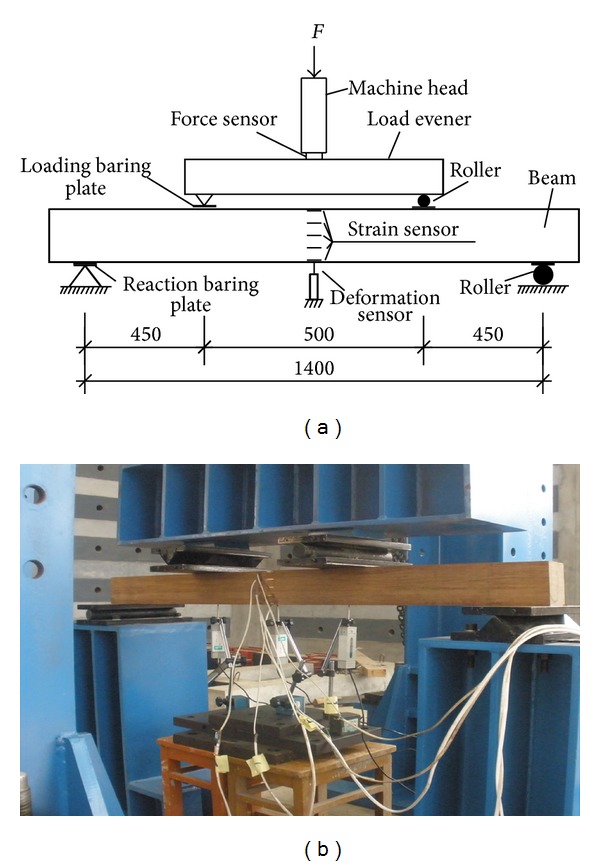
Test setup and photo of test.

The 13 PSB beams were tested to investigate their bending performances. The cross sections of the beams were 80 × 110 mm^2^, and the lengths of them were 1560 mm. 5 PSB beams were fabricated from the bottom bamboo, 4 PSB beams were fabricated from the middle bamboo, and 4 PSB beams were fabricated from the top bamboo. Because there is no standard test method for the structural members of bamboo composites, the dimensions of specimens and the test setup were designed referring to ASTM D198-02 [7]. All spans of the beams were taken as 1400 mm. 450 mm long half-shear-span was taken into account, a beam simply supported subjected to a two-point load at the third span, referring to Figure 1. No lateral support was needed to prevent the beams from lateral instability because the depth-to-width ratio was less than 3. Five electrical strain gauges parallel to the longitudinal direction of the beam were uniformly and symmetrically adhered over the side surface in the middle span. The sixth was at the bottom. Three deformation sensors were installed under the middle span of the beam and the spot of load to measure the deflection there. Total load on the beam was symmetrically and monotonously applied at two points equidistant from the reactions. The details of the test setup are shown in Figure 1. Loading speed is controlled by force at the rate of 2 kN/min while it is controlled by deflection in the middle span at the rate of 1 mm/min at a later stage till damage occurred. The total load of the beam, the strains in longitudinal direction over the side surface in middle span, and the deflection in middle span were simultaneously recorded.

### 2.3. The Damage Mechanism of PSB Beams 

#### 2.3.1. Phenomena of the Damage

**Figure 2:**
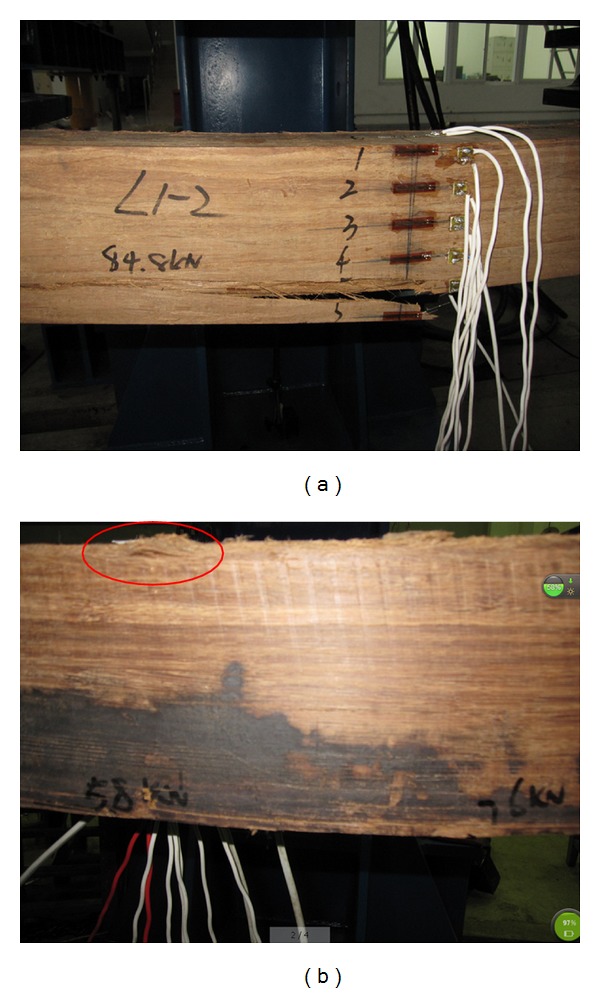
Failure modes of specimens.

The phenomena of the damage process could be summarized as follows. In the earlier stage of loading, the deflections in the middle span were linearly associated with the augmentation of load. When the load exceeded 1/3 to 1/2 of the ultimate load, some fine cracks within the moment span emerged and expanded along the parallel-to-grain direction below the neutral axis as the load increased, and bulking could be observed at the top surface of the beams in some cases. Finally the break occurred at the bottom of the beams when the loading reached the ultimate value (referring to Figure 2). The damage was almost presented in the pure moment span, which was due to bending damage.

#### 2.3.2. Mode of Failure in Bending

The bending strength of PSB is a complex problem. Bending strength depends on three main factors: the ratio of tension to compression strength of the material, nonlinear ductile behavior in the compression zone, and size-dependent brittle fracture in the tension zone. The tension strength of PSB was far greater than compression strength according to preliminary work of authors of this paper [8]. In this mode, maximum moment was associated with a brittle tension failure, but after some compression yielding had occurred. As compression yielding occurred, the neutral axis shifted toward the tension face, and tension stresses continue to increase until failure occurred as a rupture in the tension zone. The load-deflection relationship became curved after compression stresses exceed the proportional limit.

In summary, the bending damage mechanism of the beams could assume that (i) the beams were in the linear stage and could be idealized as a perfect elastic element at the beginning of loading; (ii) with the augmentation of loading, beams went into the nonlinear stage; the fibers in the top of the compressive zone, which were over the neutral axis of the cross section in the moment span, first reached the plastic state and the stress on them was gradually coming to the ultimate compressive strength from the outside extending to the inside of the beam; and (iii) the stress of the fibers in the outside of the tensile zone, which was below the neutral axis in the moment span, finally reached its ultimate (maximum) tensile strength and then broke.

## 3. Analysis of Experiments for PSB Beams

### 3.1. Strain of the Cross Section

**Figure 3:**
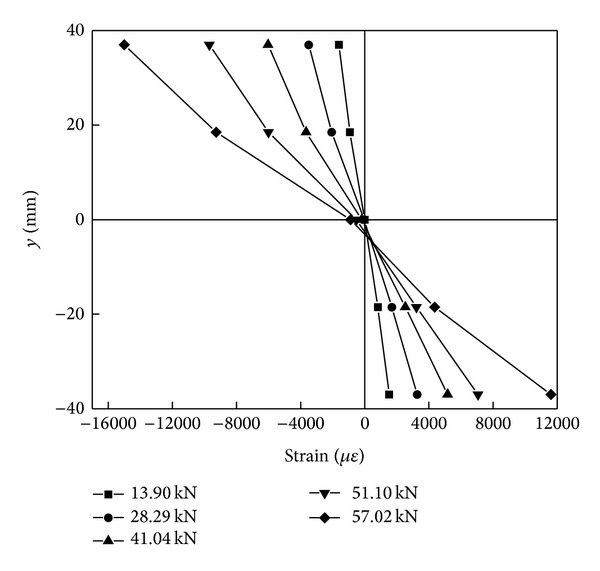
Strain distribution in parallel-to-grain direction over the depth of section.

Figure 3 showed the longitudinal strains over the bending section of a beam obtained from the experiments. It could be found that the envelope of longitudinal strains at the 5 measuring points remained approximately linear from the beginning of loading to damage. This implied that the longitudinal strain at the various points across the section was proportional to the distance from the neutral axis. Thus the plane assumption, that is, plane sections before bending remained plane after bending, was nearly corrected for the flexure of PSB beams. Therefore, the influence of the shearing's effects on the bending of the beam may be neglected. Figure 3 also showed that the neutral axis of the bending section was offsetting towards its lower part during loading process. This indicated that the compressive zone was expanding towards the lower part of the beam during the loading process because the fibers were gradually bulking from the top surface to the inside of the beam.

### 3.2. Analysis of Load-Strain of Beams

**Figure 4:**
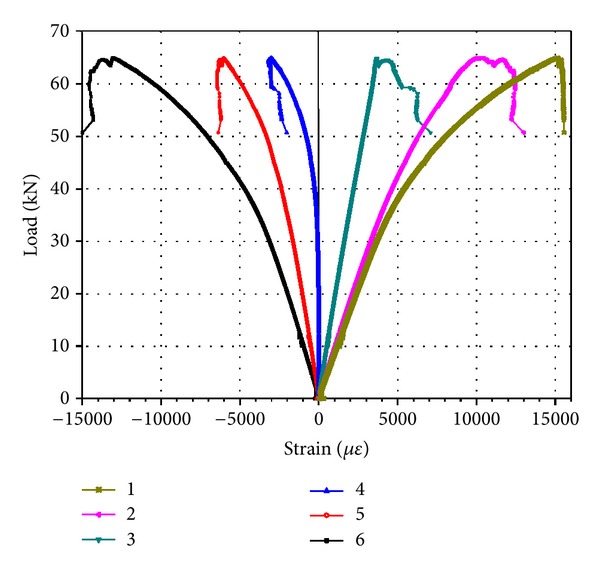
Load-strain typical curve of PSB beam.

The relationship of strain changing with load was shown in Figure 4 that tensile strain was positive and compressive strain was negative. Line 1 was the strain of the bottom of beam. The approximate linear relationship between strain and load in the beginning stage of loading could be seen in the figure. In the subsequent stage of loading, strain augmented faster than load. Strain was released suddenly in the last damage stage, so the strain of the measuring points decreased. The maximum tensile strain approaching destruction was commonly 10000 *με*~12000 *με*, as only a few specimens achieved 15000 *με*~16000 *με*; the maximum compressive strain of bamboo fiber was commonly 10000 *με*~14000 *με*, and a few specimens achieved 16000 *με*. Compressive strain was higher than the tensile strain and further showed the difference between tension and compression elastic modulus, as the tension modulus of PSB was more than the compression modulus. The change of the ultimate strain range was wide, also showing the large variability in performances of PSB.

### 3.3. Curves of the Load-Deflection of Beams

**Figure 5:**
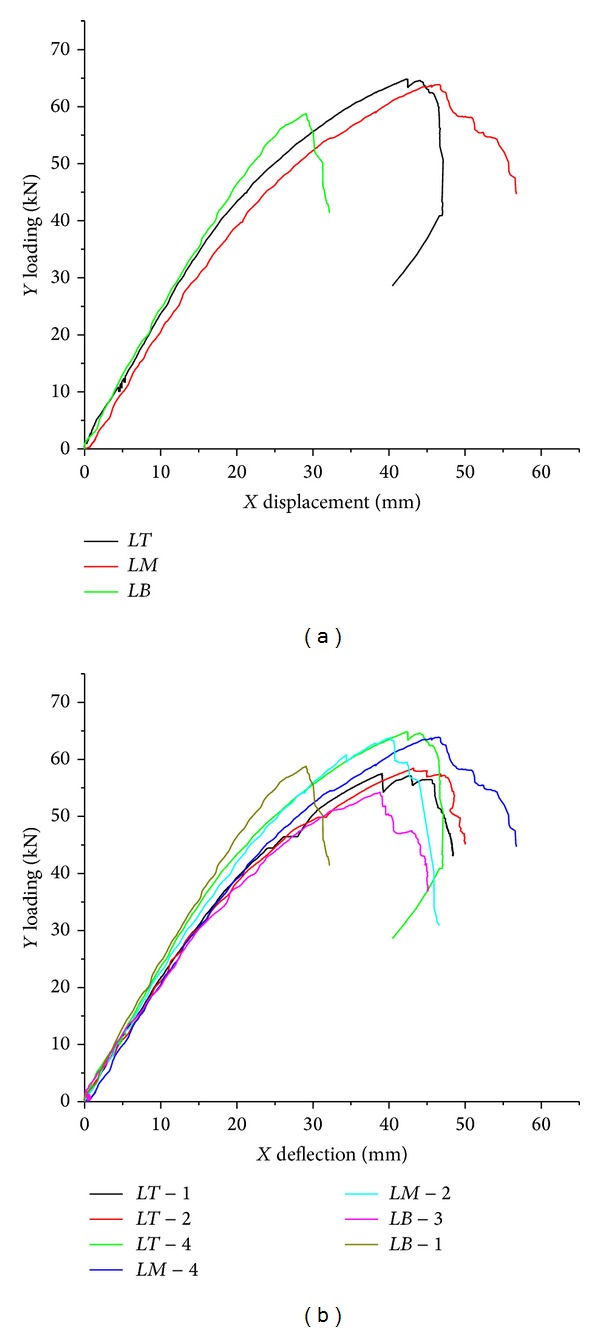
(a) Load versus midspan deflection typical curves of PSB beams the tip.mid.bottom, respectively. (b) Load versus midspan deflection curves of most specimens.

The direct results obtained from the bending tests are the load versus midspan deflection curves. Figures 5(a) and 5(b) present the curves obtained for PSB beams The linear relationship between deflections and load was shown only at the beginning of loading; the deflection-load curve began to deviate from the straight line after 1/3~1/2 of the ultimate load. The bending stiffness of the beam gradually reduced because there were some flaws and gaps distributing randomly in the bamboo fibers of the PSB beam. In the process of loading, stress in the flaws appeared as distortion, leading to its surrounding stress more than the stress limit of the fiber or agglutination; at the same time some compression yielding has occurred, then the crack was extended constantly, and stiffness decayed simultaneously. After the maximum load, bearing capacity dropped rapidly. Large nonlinear deformations were achieved and the ultimate deflections of PSB beams could reach about 1/35 span before breaking. PSB beams made from different parts of raw bamboo had different performance, referring to Figure 5(a). The mechanical performances of the PSB beams made from the bottom bamboo were inferior to that of the upper.

### 3.4. The Mechanical Performance of PSB Beams

The test result showed that tension modulus was slightly superior to the compression modulus. The load-deflection information derived from the 4-point bending tests can be analyzed using formulae for structural analysis. In this case, formulae for beams under bending loads are used. Assuming a perfect adhesion between PSB, in the proposed tests *L*/*h* = 13.2, in this case shear effects could be neglected referring to ASTM D198-02. The deflection of straight beams that are elastically stressed and have a constant cross section throughout their length could be given by the theory of mechanics of materials. And then the maximum bending stress, deflection of load, is given by

(1) σmax⁡=3FL1bh2,f=FL14Ebh3(3L2−4L12),

where *σ*_max⁡_ is the ultimate strength and *f* and *E* are deflection of midspan and Young's modulus in parallel-to-grain directions, respectively. *F* is the total beam load acting perpendicular to beam neutral axis. *b* and *h* are width and height of beam, respectively. *L* and *L*_1_ is 1400 mm and 450 mm respectively referring to Figure 1. The formula was only suitable for the linear range of load-deflection curves. Young's modulus of PSB can be given using the Hooke's law assuming a uniaxial stress state in the grain direction:

(2) E=3FL1bh2εmax⁡,

(3) E=FL14fbh3(3L2−4L12),

where *ε*_max⁡_ is maximum strain of midspan beam in the elastic stage; compression modulus was obtained by maximum strain in the compressive zone of the beam; tension modulus was acquired from maximum strain in the tensile zone of the beam. According to (2) and (3), Young's modulus was calculated by the strain collected continuously through strain gauge. The elastic stage was confirmed when the Young's modulus began to decline. Although (2) and (3) are valid for linear elastic behavior, they are also used beyond the limits of Hooke's law. When using these equations beyond elastic limit, stresses and strains can be considered as pseudostresses and pseudostrains, respectively.  Table 1 showed the mean, standard, and deviations of variation of the maximum bending stress, the Young's modulus for the tested specimens. These properties corresponded to the longitudinal direction.

The bending strength and elastic modulus of upper PSB was about 10% higher than that of the bottom referring to Table 1. According to the early mesoscopic research about bamboo by the authors in this paper, bamboo is a kind of composite composed of a vascular bundle and matrix (thin-walled cells). The mechanical performances of bamboo depended on its vascular bundle; the content of the vascular bundle in the bottom was less than that of the upper [9]. So the mechanical performances of the bottom were less than that of the upper.

## 4. Conclusions 

An overall analysis of the experimental results revealed the following.

1. Mode of failure in bending was nonlinear ductile behavior in the compression zone and size-dependent brittle fracture in the tension zone. As compression yielding occurred, the neutral axis shifted toward the tension face, and tension stresses continued to increase until failure occurred as a rupture in the tension zone. The bending damage mechanism of the PSB beams could assume that (i) the beams were in the linear stage and could be idealized as a perfect elastic element at the beginning of loading; (ii) with the augmentation of loading, beams went into the nonlinear stage, as the fibers in the top of the compressive zone firstly reached the plastic state; and (iii) the stress of the fibers in the outside of the tensile zone finally reached its ultimate tensile strength before breaking.
2. The PSB beams were accorded with the plane assumption. Therefore, the influence of the shearing effect on the bending of the beam may be ignored. Large nonlinear deformations were achieved and the ultimate deflections of PSB beams could reach about 1/35 span before breaking.
3. The tension modulus was slightly superior to the compression modulus. The mechanical performances of PSB made from the bottom raw bamboo were inferior to that of the upper. The bending strength and Young's modulus of PSB could be about 89 MPa, and 12000 MPa respectively.

## Figures and Tables

**Table 1:** Test results of specimens.

Number	*σ*_max⁡_/MPa	*E*/MPa	*E*_*t*_/MPa	*E*_*p*_/MPa
Top				
LT-1	87.81	12604	12734	12438
LT-2	96.56	12548	13029	13874
LT-3	93.07	12434	12736	12420
LT-4	88.08	12403	13792	13342

Mean	91.38 ± 3.43	12497 ± 78.75	13072 ± 359.62	13018 ± 589.5

Mid				
LM-1	95.49	12464	12733	12863
LM-2	95.95	13037	15291	14425
LM-3	86.76	13981	13365	14409
LM-4	88.17	12313	13747	12614

Mean	91.59 ± 4.13	12949 ± 560.25	13784 ± 753.5	13577 ± 839.25

Bottom				
LB-1	88.98	12112	12232	10933
LB-2	81.60	13765	13404	13506
LB-3	91.05	13639	14718	13002
LB-4	85.90	11341	11692	10083
LB-5	81.81	11882	12407	12398

Mean	85.87 ± 3.33	12548 ± 644.51	12833 ± 1091.08	11984 ± 1435.5

Total mean	89.32 ± 4.92	12656 ± 763.94	13221 ± 996.19	12793 ± 1243.6

Where *σ*_max⁡_ is the bending strength; *E* is bending Young's modulus acquired according to (3); *E*_*t*_ is tension modulus acquired according to (2); *E*_*p*_ is compression modulus. acquired according to (2).

## References

[1] Liangming Y, Zhihong J, Jianhua Y (1991). Research of recombinant bamboo board. *Journal of Zhejiang Forestry College*.

[2] Changjun F *Experimental study on axial compression member of parallel strand bamboo [Dissertation]*.

[3] Yang W, Shenxue J, Qingfang L (2010). Experimental study on flexural performance of bamboo beams. *Building and Structure*.

[4] Qingfang L, Yang W, Qisheng Z (2008). Experimental study on mechanical properties of basic components for a new anti-seismic room with bamboo engineering materials. *Research and Application of Building Materials*.

[5] Xiao Y, Ma J (2012). Fire simulation test and analysis of laminated bamboo frame building. *Construction and Building Materials*.

[6] Liqing, Kuihong W (2001). A preliminary study on the production technology of recombinant bamboo. *Artificial Board Communication*.

[7] American Society for Testing Materials

[8] Dongsheng H, Aiping Z, Yuling B (2013). Experimental and analytical study on the nonlinear Bending of Parallel Strand Bamboo Beams. *Construction and Building Materials*.

[9] Zhou A, Huang D, Li H, Su Y (2012). Hybrid approach to determine the mechanical parameters of fibers and matrixes of bamboo. *Construction and Building Materials*.

